# Specific Hepatorenal Toxicity and Cross-Species Susceptibility of Eight Representative Pesticides

**DOI:** 10.3390/toxics13110911

**Published:** 2025-10-23

**Authors:** Yue Liu, Ning Xu, Xinyu Song, Muchen Deng, Ranfeng Sun, Peilong Wang, Lidong Cao

**Affiliations:** 1 School of Tropical Agriculture and Forestry, Hainan University, Haikou 570228, China; liuyue2399@163.com (Y.L.); srf18@hainanu.edu.cn (R.S.); 2 Institute of Quality Standard and Testing Technology for Agro-Products, Chinese Academy of Agricultural Sciences, Beijing 100081, China; wangpeilong@caas.cn; 3 Institute of Plant Protection, Chinese Academy of Agricultural Sciences, Beijing 100193, China; songxinyucn@163.com (X.S.); 15541201860@163.com (M.D.)

**Keywords:** pesticide, hepatorenal toxicity, cross-species comparison, in vitro toxicology, biomarkers

## Abstract

Chronic exposure to pesticides poses significant hepatorenal toxicity risks, yet a systematic comparison of their effects across species and tissues is lacking. In this study, we systematically evaluated the cytotoxicity of eight pesticides using human (CCC-HEL-1 hepatocytes; 293T renal cells) and rodent (IAR hepatocytes; NRK renal cells) cellular models. Our results showed substantial variations in potency, with chlorothalonil exhibiting the highest toxicity (IC50 = 32.55 mg/L in 293T cells) and chlorpyrifos the lowest (IC50 = 444.5 mg/L in 293T cells). Principal component analysis revealed distinct species- and tissue-specific response patterns, highlighting the unique resistance of NRK cells. Mechanistic investigations demonstrated organ-specific biomarker alterations, such as elevated hepatic ALP and suppressed renal KIM-1. Remarkably, the MRP2 transporter exhibited tissue-specific divergence, being significantly downregulated in renal cells (all 8 pesticides, *p* < 0.005) and most hepatic cells (7/8 pesticides, *p* < 0.05), while propiconazole uniquely upregulated it in hepatocytes (1.5-fold, *p* < 0.05). Collectively, these findings offer critical mechanistic insights into pesticide-specific toxicity and cross-species susceptibility, providing valuable data to improve human health risk assessment in food safety and toxicology.

## 1. Introduction

Pesticides are indispensable tools in modern agriculture, crucial for managing pests and ensuring crop productivity. However, their extensive use has raised significant concerns regarding environmental and human health impacts [1]. Globally, over 1000 active pesticide ingredients are registered, with annual usage exceeding 5.6 billion pounds [2]. While this widespread application supports food security, it also contributes to environmental contamination [3]. Research indicates that approximately 60% of applied pesticides enter the environment through volatilization, runoff, or leaching [4]. Following metabolic transformation in the environment, these pesticide residues not only disrupt the physiological functions of non-target organisms but may also threaten ecosystem stability through food chain bioaccumulation [5]. Chronic exposure to pesticide residues has been associated with endocrine disruption [6], neurotoxicity [7] and reproductive toxicity [8] along with metabolic disorders including obesity and diabetes [9]. These findings highlight a critical challenge for sustainable agricultural development [10,11]. The dual aspects of pesticide benefits and environmental health impacts necessitate a balanced approach to pesticide management and regulation.

Among the myriad of pesticides, triazole fungicides and organophosphate insecticides have garnered significant attention due to their extensive application and persistent environmental residues [12]. Triazole fungicides, known for their endocrine-disrupting properties, exert their effects by inhibiting cytochrome P450 enzymes involved in steroidogenesis. Organosphate insecticides, particularly chlorpyrifos, have been widely employed for pest control across various agricultural products, including fruits, vegetables, and grains [13]. Environmental monitoring data from Shanxi Province have identified chlorpyrifos as the most frequently detected pesticide residue in agricultural soils, highlighting its environmental prevalence [14]. Another notable contaminant Chlorothalonil, a broad-spectrum organochlorine fungicide. Its extensive use in global agriculture is evidenced by its ranking as the tenth most common active ingredient in the U.S. agricultural sector and the second most widely used pesticide in industrial, commercial, and governmental applications [15]. Furthermore, emerging fungicides like pyrimethanil are increasingly concerning due to their environmental mobility and persistence. This broad-spectrum systemic fungicide, commonly used to control Botrytis cinerea (gray mold) across various crops, has been detected in aquatic environments at concentrations ranging from744 to 9591 ng/L in agro-industrial wastewater [16]. The environmental persistence and bioaccumulation potential of these pesticides, despite their agricultural efficacy, have raised substantial concerns regarding their detrimental impacts on non-target organisms, human health, and ecosystem integrity. Supplementary Material Table S1 provides a comprehensive overview of the acute toxicity and acceptable daily intakeADI) values for the eight pesticides studied, offering valuable insights into their potential health impacts and regulatory thresholds.

Currently, China faces significant gaps in the systematic integration and comparative analysis of pesticide toxicity data. While various in vitro models [17], including rat [18] and zebrafish [19] systems [20], have been established for assessing acute and chronic pesticide toxicity, mechanistic understanding of toxicological effects remains insufficient. In response, cell culture-based in vitro approaches are gaining prominence in modern toxicology research due to their distinct advantages over traditional animal models. These methods offer higher standardization in experimental protocols, significantly faster turnaround times, and compatibility with high-throughput screening (HTS) platforms. Such technical merits have made in vitro cytotoxicity assays a widely adopted tool in chemical safety assessment, particularly for preliminary toxicity screening and mechanistic studies of pesticides. A representative example is a study in which researchers evaluated the cytotoxicity of 108 pesticides across 10 human-derived cell types. The results demonstrated a correlation between in vitro data from SH-SY5Y cells and in vivo safety guidelines. This finding can assist field investigators in identifying and prioritizing areas of concern, as well as environmental media affected by complex pesticide mixtures at contaminated sites [21].

The liver, as the primary organ for metabolizing and detoxifying foreign substances, is particularly vulnerable to pesticide-induced damage [22]. The hepatic microsomal cytochrome P450 (CYP450) enzyme system plays a central role in metabolizing pesticides and other toxic chemicals. However, this biotransformation process may simultaneously induce two critical pathological mechanisms: functional disruption of the CYP450 enzyme system and interference with mitochondrial energy metabolism pathways [23]. These molecular disturbances progressively lead to hepatocellular damage, ultimately manifesting as varying degrees of hepatotoxicity. The traditional liver biomarkers—alanine aminotransferase (ALT), aspartate aminotransferase (AST), and alkaline phosphatase (ALP)—are widely used to assess hepatic injury [24]. Combining these three biomarkers enhances the specificity and sensitivity in evaluating chemically induced liver damage [25]. The kidneys, as vital excretory organs, play a crucial role in waste elimination and drug metabolism [26], while simultaneously serving as a primary target for pesticide toxicological effects. Growing epidemiological evidence has established significant associations between pesticide exposure and the development of renal diseases. Of particular concern is the potential of certain pesticides to promote chronic kidney disease (CKD) progression by inducing subclinical renal injury [27]. Plasma kidney injury molecule-1 (KIM-1) has been identified as a sensitive biomarker for proximal tubule injury [28]. Its expression increases during the early stages of renal damage and remains elevated throughout subsequent cellular injury, proliferation, and regeneration phases. This characteristic makes KIM-1 particularly valuable for detecting early acute kidney injury following exposure to nephrotoxic chemicals or medications [29]. The renal excretion of drugs is primarily mediated by drug transporters, among which Multidrug Resistance-associated Protein 2 (MRP2) plays a crucial role [30]. As a member of the ATP-binding cassette (ABC) transporter family, specifically the multidrug resistance-associated protein (MRP) subfamily, MRP2 is predominantly expressed on cellular membranes and widely distributed in various human tissues including the liver, kidneys, intestines, and brain.

This study investigated eight agriculturally important pesticides with high residue risks, comprising five triazole fungicides (myclobutanil, tebuconazole, difenoconazole, propiconazole, and triadimefon), one organophosphate insecticide (chlorpyrifos), one anilinopyrimidine fungicide (pyrimethanil), and one chloronitrile fungicide (chlorothalonil). We established an in vitro toxicity assessment system using four mammalian cell lines: human hepatocyte line (CCC-HEL-1), human 293T renal cells, rat liver epithelial cells (IAR), and rat kidney epithelial cells (NRK-52E). The selection of these two cell models was based on their distinct biological characteristics and experimental advantages, which align with the specific research objectives of this study. CCC-HEC-1 cells, derived from human embryonic liver tissue, were chosen for their retention of key hepatic metabolic functions, making them an ideal in vitro model for assessing pesticide-induced hepatotoxicity. Their ability to mimic human liver physiology provides reliable insights into organ-specific toxic effects. 293T cells were selected due to their high transfection efficiency and stable expression of exogenous genes, which are essential for validating candidate biomarkers and investigating molecular regulatory mechanisms. By integrating these two models, this research aims to comprehensively elucidate the biological effects of pesticides from the dual perspectives of organ-specific toxicity assessment and molecular mechanism analysis.

Rats, widely used in toxicological studies due to their physiological and metabolic similarities to humans, provide reliable data for extrapolating toxicological effects to humans [31,32]. Comparative pharmacokinetic analysis of the herbicide dicamba in rats and humans established a data-derived extrapolation factor (DDEF), demonstrating that humans exhibit lower toxicity sensitivity to HPPD inhibitors than rats, highlighting the utility of data-driven approaches in improving pesticide risk assessment accuracy [33]. Human cell lines further enhance the direct relevance of findings for human health risk assessment [34].

This experimental design enabled systematic comparison of cytotoxic effects across different pesticide classes and cell types, with the dual objectives of elucidating the cellular mechanisms underlying pesticide toxicity while generating robust scientific data to inform food safety standards and human health risk evaluations. The comprehensive approach not only facilitates identification of high-risk pesticides but also reveals potential species-specific toxicity patterns, thereby contributing to the development of more accurate predictive models for assessing human health impacts.

## 2. Materials and Methods

### 2.1. Reagents and Instruments

To investigate the toxicological effects of pesticides, we employed a panel of four mammalian cell lines obtained from the Institute of Basic Medical Sciences, Chinese Academy of Medical Sciences. The selection was designed to provide both physiologically relevant and mechanistically tractable models. Human liver CCC-HEL-1 cells were used to model hepatic toxicity due to their retention of characteristic drug-metabolizing enzyme activities. In parallel, Human embryonic kidney 293T cells were utilized for their high transfection efficiency, enabling the functional screening and mechanistic study of candidate biomarkers. To provide comparative data, Normal Rat Kidney (NRK) cells and Rat liver epithelial (IAR) cells were also included. The study involved eight pesticide standards with a purity of ≥98% (myclobutanil, tebuconazole, difenoconazole, propiconazole, triadimefon, chlorpyrifos, pyrimethanil, and chlorothalonil), which were sourced from Tianjin Alta Scientific Co., Ltd. (Tianjin, China) and Beijing Qincheng Yixin Technology Co., Ltd. (Beijing, China) (detailed in Table 1).

High-glucose DMEM, MEM and 0.01 mmol/L PBS (pH 7.4) (Invitrogen, Waltham, MA, USA); 0.25% Trypsin-EDTA (0.25%) (Gibco, Waltham, MA, USA) and fetal bovine serum (FBS) (Corning, Corning, NY, USA), Cell viability assay: CCK-8 kit (Beijing BioRab Biotechnology Co., Ltd., Beijing, China), Dimethyl sulfoxide (DMSO; Beijing Yanqiwan Biotechnology Co., Ltd., Beijing, China), 1300 Series A2 Biosafety Cabinet (Thermo Fisher Scientific, Waltham, MA, USA), VACUSAFE comfort vacuum pump (Eppendorf AG, Hamburg, Germany), D1008 microcentrifuge (SCILOGEX LLC, Rocky Hill, CT, USA).

### 2.2. Cell Culture and Chemicals

Human embryonic kidney 293T (HEK293T) cells and rat renal NRK cells were maintained in high-glucose DMEM supplemented with 10% fetal bovine serum (FBS) and 1% penicillin–streptomycin at 37 °C under 5% CO_2_ until reaching 70–80%. Human liver CCC-HEL-1 cells were cultured under identical conditions but with 20% FBS, while rat normal hepatocytes were grown in high-glucose MEM containing 20% FBS and 1% penicillin–streptomycin. Stock solutions (500 g/L) of eight pesticide standards were prepared in dimethyl sulfoxide (DMSO) and stored at −20 °C. Working concentrations were freshly diluted in serum-containing culture medium prior to each experiment.

### 2.3. Cell Culture and Exposure

The cytotoxicity of eight pesticides on four mammalian cell lines was assessed using the Cell Counting Kit-8 (CCK-8) assay. Initially, stock solutions of each pesticide were prepared by dissolving the compound in dimethyl sulfoxide (DMSO) to a final concentration of 500 g/L. For the assay, cells were seeded into 96-well plates at a density of 2 × 10^4^ cells per well in 100 µL of medium and incubated for 24 h at 37 °C under a 5% CO_2_ atmosphere to allow for attachment. Subsequently, the culture medium was replaced with fresh medium containing the pesticides at a series of final concentrations (0, 10, 100, 200, 400, 800, 1000, and 2500 mg/L). Pesticide working concentrations were prepared through serial dilution of the 500 g/L DMSO stock solution, resulting in variable final DMSO concentrations across treatment groups (ranging from 0.002% to 0.5% *v*/*v*). A media-only blank (no cells) was included for background absorbance correction, and a solvent control (0.1% DMSO, no pesticide) was used to account for any vehicle effects. All conditions were performed in five replicates. A separate experiment confirmed that none of the DMSO concentrations used (0.1–1% *v*/*v*) significantly affected cell viability compared to the DMSO-free control. At DMSO concentrations ranging from 0.1% to 0.2%, the viability of all four cell lines remained stable above 80%. At a 1% DMSO concentration, the viability of all four cell types was around 50% or higher (Supplementary Figure S5). Following 24 h of exposure, 10 µL of CCK-8 reagent was added to each well. Absorbance Measurement: After an additional 1 h of incubation in the dark, the optical density (OD) at 450 nm was recorded using a microplate reader. Cell viability was calculated by comparing the OD values of treated groups with those of the blank control. Although some test concentrations exceeded the water solubility of the pesticides, the combination of DMSO and serum in the culture medium maintained a uniform exposure system. No precipitation was observed during the experiment, ensuring that the measured effects are attributed to the bioavailable fraction of the compound.

### 2.4. ELISA for Hepatorenal Toxicity Biomarkers

The levels of hepatotoxicity biomarkers (ALT, AST, ALP) and nephrotoxicity biomarkers (KIM-1, LDH) in cell culture supernatant were quantified using commercially available ELISA kits (Cloud-Clone Corp., Wuhan, China). 100 μL of the supernatant was added to the pre-coated wells and incubated according to the manufacturer’s protocol (five replicates). After washing, the appropriate detection antibody and substrate were added, and the absorbance was measured at 450 nm using microplate reader. The concentrations of the biomarkers were determined based on standard curves.

### 2.5. Immunofluorescence Staining

Cells were seeded at a density of 1 × 10^5^ cells per well in immunofluorescence-specific glass-bottom dishes and allowed to adhere for 24 h. After attachment, cells were treated with eight pesticides at their respective half-maximal lethal concentrations (IC_50_) and incubated for an additional 24 h at 37 °C in a humidified 5% CO_2_ atmosphere. Following treatment, cells were fixed with 4% paraformaldehyde for 15 min at room temperature and permeabilized with 0.1% Triton X-100 for 10 min. Non-specific binding was blocked with 5% bovine serum albumin (BSA) for 1 h at room temperature. Cells were then incubated with primary antibody against MRP2 (1:300 dilution) overnight at 4 °C. After three washes with PBS, cells were treated with secondary antibody (rabbit anti-IgG, 1:600 dilution) for 1 h at room temperature and washed three times with PBS. Anti-MRP2 antibody (Abcam, ab172630) and Alexa Fluor^®^ 488-conjugated anti-rabbit IgG secondary antibody (Cell Signaling Technology, Danvers, MA, USA, 4412) were used for immunofluorescence staining. Nuclei were counterstained with DAPI Staining Solution (C1005, 10 mL) (Beyotime Biotechnology, Shanghai, China). Fluorescent images were captured using a 10× inverted fluorescence microscope. Quantitative analysis of fluorescence intensity was performed using ImageJ software 2.1.0 (National Institutes of Health, Bethesda, MD, USA).

### 2.6. Statistical Analysis

Statistical analysis was performed using SPSS 26.0 software (IBM Corp., Armonk, NY, USA), with data presented as mean ± standard deviation. GraphPad Prism 9.5 (GraphPad Software, Inc., Boston, MA, USA) was employed for data calculation, organization, and figure generation.

## 3. Results

### 3.1. Analysis of Pesticide Toxicity Variations

**Figure 1:**
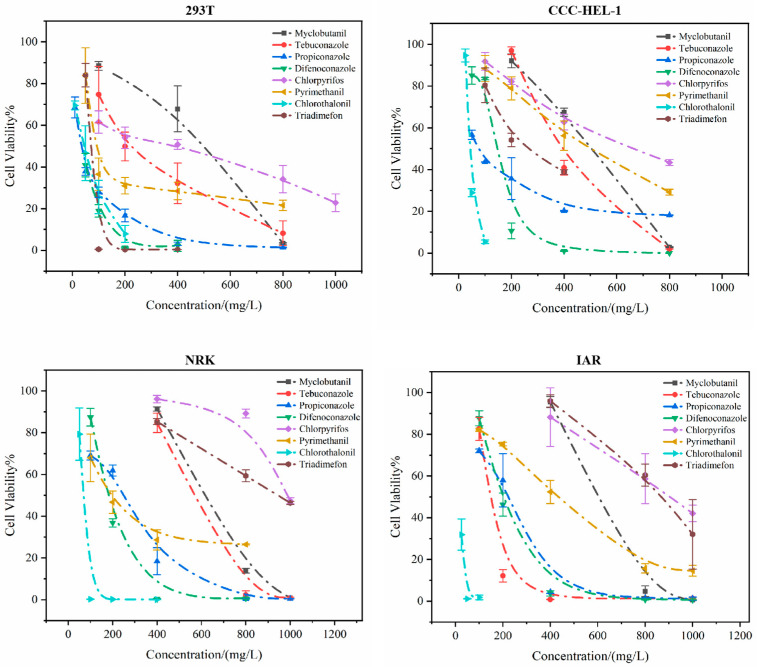
Effects of eight pesticides on cell viability in four cell lines.

Systematic evaluation of the cytotoxic effects of eight pesticides on four cell lines using the CCK-8 assay revealed significant differences in their impacts on cell viability (Supplementary Material, Figures S1–S4), all of which exhibited a dose-dependent relationship—cell survival rates decreased with increasing pesticide concentrations (Figure 1). Notably, chlorothalonil displayed the strongest cytotoxicity in three cell lines (CCC-HEL-1 hepatocytes, NRK renal cells, and IAR hepatocytes). Although propiconazole showed higher toxicity than chlorothalonil in 293T cells, chlorothalonil still exhibited considerable toxicity (IC_50_ = 32.55 mg/L) and demonstrated strong cytotoxicity across all four cell lines. In contrast, chlorpyrifos consistently exhibited the lowest toxicity in CCC-HEL-1, NRK, and IAR cells. Its IC_50_ value in 293T cells (444.5 mg/L) was the second highest (indicating lower toxicity), following myclobutanil (IC_50_ = 458.5 mg/L), suggesting mild cytotoxicity across all tests.

### 3.2. Cross-Cell Line and Species-Specific Toxicity Profiling

**Figure 2:**
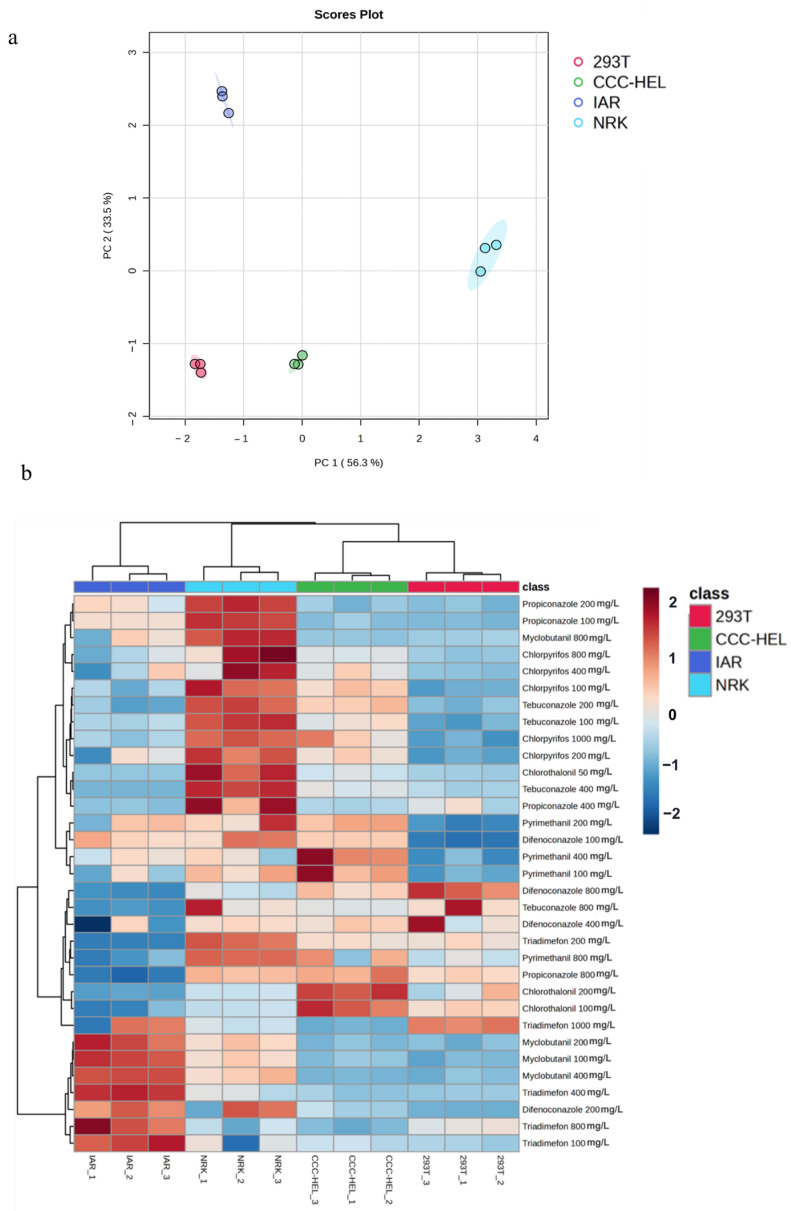
Multivariate analysis of cytotoxic responses across drug treatments. (**a**) Principal component analysis (PCA) of cytotoxicity profiles induced by different drug treatments in four cell lines. (**b**) Heatmap analysis of cytotoxicity responses (OD values) in four cell lines exposed to multiple drugs at varying concentrations.

The cytotoxic effects of eight pesticides on four distinct cell lines were systematically evaluated using CCK-8 assay, revealing significant variations in cellular responses. Principal component analysis (PCA) demonstrated clear separation among cell types in response to different treatment conditions, with the first two principal components (PC1: 56.3 PC2: 33.5%) accounting for 89.8% of the total variance (Figure 2a). The two-dimensional PCA plot revealed distinct clustering patterns: 293T (human kidney cells) occupied the lower-left quadrant, CCC-HEL (human liver cells) were centrally positioned with a leftward bias, IAR (mouse liver cells) were distributed in the upper-left region, while NRK (mouse kidney cells) were distinctly separated in the right quadrant. Notably, NRK cells exhibited significant separation along the PC1 axis, suggesting unique drug sensitivity compared to other cell lines. The analysis revealed that cellular responses to drug toxicity were influenced both species origin (human vs. mouse) and tissue type (kidney vs. liver), with particularly distinct responses observed between kidney-derived cells from different species.

Heatmap analysis of z-score normalized OD values revealed distinct cytotoxicity profiles among the four cell lines (293T, CCC-HEL, IAR, NRK) under varying pesticide concentrations, with color gradients indicating response intensity (red: high viability/low toxicity; blue: low viability/high toxicity) (Figure 2b). The analysis demonstrated clear species- and tissue-specific toxicity patterns: 293T cells (human kidney) formed a tightly clustered red group, indicating consistent responses across treatments; CCC-HEL (human liver) and IAR (mouse liver) exhibited adjacent but distinct green and blue clusters, respectively; while NRK cells (rat kidney, cyan) showed a unique response pattern, clearly separated from the other cell types.

The clustering analysis of pesticide cytotoxicity revealed distinct patterns based on concentration and cell type specificity. High concentrations of certain pesticides, including Myclobutanil, Triadimefon, and Difenoconazole, demonstrated strong cytotoxic effects across most cell types, as indicated by deep blue coloration in the heatmap. In contrast, Chlorothalonil and Tebuconazole exhibited relatively lower cytotoxicity at specific concentrations, represented by red or light blue regions. Notably, differential cellular responses to identical pesticide treatments were observed, highlighting cell type-specific sensitivity to chemical exposure. These findings underscore the importance of considering both concentration-dependent effects and cellular specificity when evaluating pesticides.

### 3.3. Comparative Analysis of Hepatotoxicity and Nephrotoxicity Biomarkers

**Figure 3:**
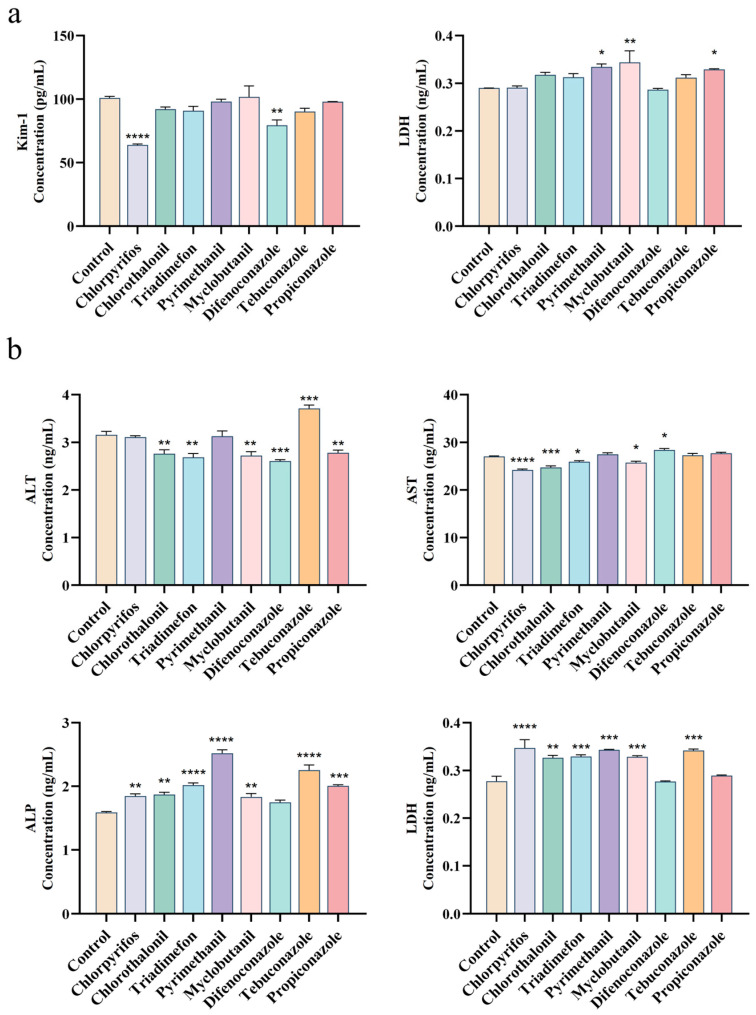
Cytotoxicity profiling of eight pesticide in human cell lines. (**a**) Expression of toxicity biomarkers in 293T renal epithelial cells following 24 h exposure to pesticide at their median lethal concentrations. Cytokine levels in cell culture supernatants were quantified by ELISA. (**b**) Corresponding biomarker expression in CCC-HEL-1 hepatocytes under identical treatment conditions. Statistical significance: * *p* < 0.05; ** *p* < 0.005; *** *p* = 0.0001; **** *p* < 0.0001. n ≥ 3.

Exposure of 293T renal cells to various pesticides for 24 h revealed distinct patterns of cellular injury markers (Figure 3). LDH release assays showed that pyrimethanil and propiconazole significantly increased LDH levels (*p* < 0.05), while myclobutanil induced an even more pronounced effect (*p* < 0.01). In contrast, most other pesticides did not significantly alter LDH release (*p* > 0.05). Analysis of KIM-1 expression demonstrated that chlorpyrifos (*p* < 0.0001) and difenoconazole (*p*< 0.001) significantly decreased this renal injury marker compared to controls. The majority of tested pesticides showed no significant effect on KIM-1 expression (*p* > 0.05).

Hepatic cells exhibited greater sensitivity to pesticide exposure. Chlorpyrifos induced the most dramatic LDH elevation (*p* < 0.0001), followed by triadimefon, myclobutanil, pyrimethanil, and tebuconazole (*p* < 0.001). Liver function biomarkers revealed compound-specific patterns: ALT: Tebuconazole caused significant elevation (*p* < 0.001); AST: Difenoconazole increased levels (*p* < 0.05) while chlorpyrifos (*p* < 0.0001) and chlorothalonil (*p* < 0.001) showed marked suppression; ALP: Most pesticides induced substantial increases, particularly tebuconazole, pyrimethanil, and triadimefon (*p* < 0.0001), suggesting potential cholestatic injury through bile acid transporter disruption. These results demonstrate distinct organ-specific toxicity profiles, with hepatic cells showing broader sensitivity to pesticide-induced damage across multiple biomarkers of cellular injury and dysfunction. The differential biomarker responses suggest varying mechanisms of toxicity among pesticide classes.

### 3.4. Multidrug Resistance-Associated Protein 2 (MRP2) Protein Expression Analysis in Liver and Kidney Cells Following Pesticide Exposure

**Figure 4:**
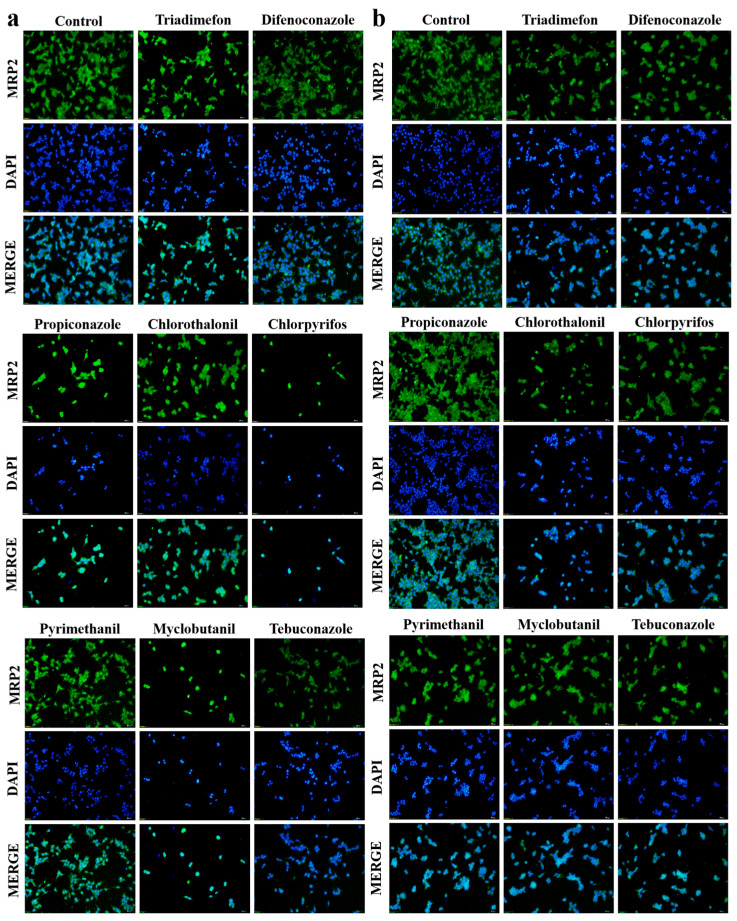
Immunofluorescence analysis of MRP2 in 293T (**a**) and CCC-HEL-1 (**b**) cells following a 24 h exposure to eight pesticides. The green fluorescence signal indicates the localization and expression level of endogenous MRP2. Green: MRP2. Blue: DAPI.

**Figure 5:**
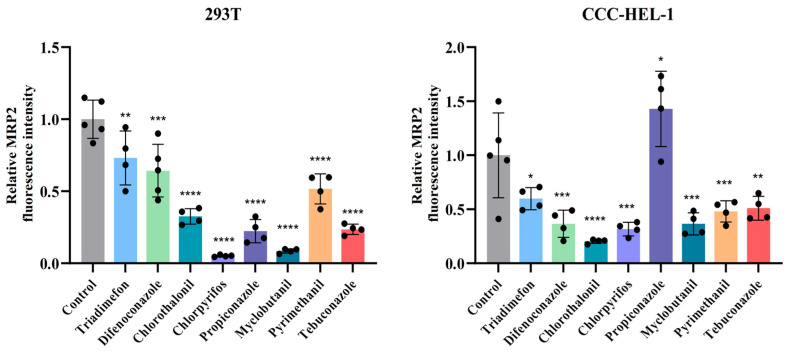
Modulation of MRP2 protein expression by eight pesticides in human hepatic and renal cells. CCC-HEL-1and 293T renal cells were exposed to pesticides at their respective IC_50_ concentrations for 24 h. MRP2 protein levels were quantified by IF. Statistical significance: * *p* < 0.05; ** *p* < 0.005; *** *p* = 0.0001; **** *p* < 0.0001. n ≥ 4.

Quantitative analysis revealed significant pesticide-induced modulation of MRP2 transporter expression in both renal and hepatic cell lines (Figure 4). In 293T renal cells, 24 h pesticide exposure caused statistically significant downregulation of MRP2 protein levels compared to untreated controls (*p* < 0.005), with chlorpyrifos and myclobutanil demonstrating the most pronounced effect. Conversely, CCC-HEL-1 hepatic cells exhibited differential responses: seven of the eight tested pesticides significantly decreased MRP2 expression (*p* < 0.05), while propiconazole treatment unexpectedly upregulated MRP2 levels by 1.5-fold relative to controls (*p* < 0.05) (Figure 5). This bidirectional regulation suggests tissue-specific mechanisms of pesticide action on transporter expression.

## 4. Discussion

This study investigated toxicity of eight pesticides to human and rodent hepatocytes and renal cells, revealing concentration-dependent cytotoxicity across all cell types. It is noteworthy that the IC_50_ values reported here were determined in an in vitro system using DMSO as a vehicle and exceed the water solubility of several compounds. These values are crucial for comparative hazard ranking of pesticides under these standardized conditions. Future studies using environmentally relevant concentrations are necessary to assess risks at typical exposure levels. Triazole pesticides, a significant class of agricultural fungicides, pose health risks through various exposure routes, including ingestion, inhalation, and dermal contact [35]. Our toxicological evaluation revealed that triazole-class pesticides exhibited moderate cytotoxicity across all four cell lines, with tebuconazole displaying preferential accumulation in liver tissue compared to kidney and urine [36], which aligns with its observed hepatotoxicity [37]. Principal component analysis (PCA) revealed distinct clustering patterns between human (293T, CCC-HEL) and rodent (IAR, NRK) cell lines in response to pesticide exposure (Figure 3). Hepatocytes (CCC-HEL, IAR) exhibited greater sensitivity to metabolically activated toxins, likely due to their enhanced drug-metabolizing capacity, while renal cells (293T, NRK) showed differential responses to excretion-related toxicants. Notably, NRK cells (rat kidney) formed a unique cluster in PCA plots and demonstrated consistently higher viability (OD values) in heatmap analysis, suggesting intrinsic pesticide tolerance. This finding highlights the potential of NRK cells as a model for studying drug resistance mechanisms, as supported by existing research utilizing NRK cells to investigate the protective effects of Tebuconazole metabolite standard 1 (TBMS1) against kidney ischemia–reperfusion injury [38] and the effects of piperazine derivatives on epithelial cell monolayer permeability [39].

Chlorothalonil, a broad-spectrum fungicide widely used in vegetable disease control, exhibits concerning bioaccumulation potential through environmental media and food chains [40]. Although chlorothalonil exhibits low acute toxicity in vivo, its known bioaccumulation potential suggests that chronic exposure could lead to high local concentrations in tissues, causing cellular damage. Our in vitro results, showing ~50% mortality at 50 mg/L, support this hypothesis. Therefore, the potent in vitro toxicity does not contradict the low in vivo acute toxicity; instead, it complements it by revealing a significant risk of localized cellular damage from chronic exposure, a risk not captured by acute systemic tests. In mammalian systems, chlorothalonil disrupts the liver–gut axis, leading to hepatic glucose metabolism dysregulation, suppressed lipoprotein lipase activity, and elevated liver function markers, including ALT and AST [41]. In aquatic organisms, it activates cytochrome P450 in kidney cells, triggering oxidative stress that downregulates the miR-15a/BCL2-A20 signaling axis, promoting cellular apoptosis and necrosis [42]. These findings collectively demonstrate that chlorothalonil exerts toxicity across diverse biological systems through distinct yet mechanistically linked pathways, emphasizing the need for comprehensive risk assessments beyond acute evaluations, particularly given its environmental persistence and bioaccumulative potential. Chlorpyrifos, a widely used organophosphorus pesticide, primarily exerts toxicity through irreversible inhibition of acetylcholinesterase (AChE) in cholinergic neurons, leading to acetylcholine accumulation, oxidative stress, inflammatory responses that damage nervous system function and peripheral organs [43]. It also exhibits significant reproductive toxicity, with high-concentration exposure reducing porcine trophoblast (pTr) cell viability and impairing fetal development in pregnant mice, as evidenced by decreased maternal body weight, placental weight, and fetal weight [44]. Interestingly, chlorpyrifos demonstrated minimal direct cytotoxicity, showing the highest IC50 values in several cell lines (CCC-HEL-1, NRK, IAR, 293T). This observation is consistent with the well-established tissue-specific toxicity of chlorpyrifos, which primarily targets the nervous system. Consequently, the non-neuronal cell models used in this study are likely inherently less susceptible to its toxic effects. Upon closer examination, a more subtle yet potentially insidious effect of chlorpyrifos emerged. Despite its mild cytotoxicity, chlorpyrifos caused a statistically significant downregulation of the kidney injury marker KIM-1 (*p* < 0.0001). This is an unexpected finding, as KIM-1 expression is typically upregulated in response to renal tubular epithelial cell damage. This paradoxical result suggests that chlorpyrifos may not injure the kidney through a typical, necrosis-inducing pathway. Instead, it might interfere with KIM-1-associated cellular repair or signaling pathways, thereby masking or altering the canonical damage response. Consistent with this disruptive effect, chlorpyrifos also demonstrated the most pronounced downregulation of MRP2 protein levels in 293T renal cells. MRP2 is a critical efflux transporter responsible for cellular detoxification. Its reduced expression would impair the cell’s ability to clear toxic substances, potentially leading to the intracellular accumulation of harmful compounds. This mechanism aligns with previously reported findings that chlorpyrifos can induce reactive oxygen species (ROS) generation, reduce mitochondrial membrane potential, and promote apoptosis [45]. Such sub-cellular damage, while insufficient to cause widespread cell death within 24 h (thus explaining the high IC50 value), is sufficient to disrupt the normal function of key proteins.

In summary, our results, combined with the existing literature, paint a more complex toxicological landscape for chlorpyrifos. While its direct cytotoxic potency is limited in certain cell types, it can significantly disrupt critical cellular pathways by downregulating key proteins like KIM-1 and MRP2. This implies that risk assessment for chlorpyrifos should not be confined to acute toxicity or AChE inhibition alone. It is crucial to also consider its potential for sub-chronic interference with specific cellular functions, such as injury response and detoxification, which may contribute to long-term health consequences.

Our study reveals significant interspecies differences in pesticide sensitivity between humans and rats, primarily driven by metabolic variations. Human cells exhibit greater pesticide sensitivity due to distinct metabolic pathways, including differential enzyme activities, metabolite profiles, and excretion routes [46,47]. Comparative analysis demonstrates distinct CYP450 expression patterns between species. While rat systems predominantly utilize CYP1A2, CYP3A2, and male-biased CYP2D1 isoforms [48], human HepG2 cells employ a more complex metabolic network centered on CYP2D6 with synergistic support from CYP1A2, 2C19, and 3A4 [49]. These differences translate to functionally significant outcomes, as seen in species-dependent clearance rates mediated by CYP3A4 (human) versus CYP3A2 (rat) [50]. Similarly, deoxyshikonin shows stronger inhibition of human CYP families compared to rat isoforms [51]. The metabolic divergence extends beyond phase I metabolism to fundamental physiological differences. Human systems preferentially conjugate bile acids with glycine, contrasting with the unconjugated forms dominant in rodent models [52]. These variations often lead to discordant results between in vitro predictions and in vivo outcomes, particularly for lipophilic compounds [53]. These findings collectively emphasize the necessity of accounting for intrinsic interspecies metabolic variations when evaluating pesticide safety profiles. While rodent models provide valuable preliminary data, direct extrapolation to human systems requires careful validation due to fundamental differences in detoxification pathways [54]. The evidence strongly supports the development of more sophisticated predictive models that explicitly incorporate species-specific metabolic parameters for accurate human risk assessment Such models would aid chemical risk assessment by reducing animal testing needs while improving human relevance [55].

Lactate dehydrogenase (LDH), an enzyme found in virtually all cells, is released into the serum upon cell membrane damage, making it a sensitive biomarker for cytotoxicity [56]. This study assessed the cytotoxicity of various pesticides in liver and kidney cells by measuring LDH release. In hepatic CCC-HEL-1 cells, 24 h exposure to most pesticides resulted in significant membrane damage and a consequent increase in LDH levels. This observation aligns with previous reports that myclobutanil decreases the viability of HepG2 cells while upregulating LDH expression [57]. In renal 293T cells, the absence of significant LDH release following 24 h pesticide exposure (*p* > 0.05) suggests that the cytotoxic mechanisms of these compounds likely do not involve acute plasma membrane disruption. Notably, three pesticides did elicit a significant response. Pyrimethanil, propiconazole, and myclobutanil all significantly elevated LDH release, demonstrating their capacity to compromise membrane integrity and induce necrotic-like cell death. In stark contrast, chlorpyrifos and difenoconazole produced a paradoxical and significant decrease in LDH release (*p* < 0.0001 and *p* < 0.001, respectively). This phenomenon could be explained by subtoxic concentrations activating cellular stress pathways that transiently enhance membrane stability or by the induction of early apoptosis, a non-lytic process that would not be detected by the LDH assay at this time point [58]. Collectively, these results suggest that while most pesticides do not cause acute membrane rupture in 293T cells, they may still exert potent, non-lytic cytotoxic effects. Therefore, further investigation is warranted. Future studies should extend the exposure duration and incorporate assays for apoptosis and other forms of programmed cell death to provide a more comprehensive understanding of the toxicological interactions between these pesticides and renal cells.

Analysis of hepatic enzymes (ALT, ALP, and AST) revealed compound-specific patterns hepatotoxicity. Pyrimethanil exposure resulted in isolated ALP elevation with normal ALT levels, a biomarker profile characteristic of cholestatic liver injury, consistent with bile duct obstruction or impaired bile excretion [59]. Tebuconazole treatment caused concurrent elevations of both ALT and ALP, suggesting mixed hepatocellular and cholestatic injury, indicative of extensive hepatic damage involving hepatocyte membranes and biliary structures [60]. In contrast, difenoconazole treatment led to elevated AST levels accompanied by reduced ALT activity. Since AST is primarily released during mitochondrial membrane disruption, this pattern suggests specific impairment of mitochondrial integrity, while the decrease in ALT may result from suppressed synthesis due to depletion or altered release kinetics [61]. Epidemiological studies support these findings, showing gender-specific liver dysfunction in Thai farmers chronically exposed to pesticides, with males exhibiting elevated AST and females showing increased ALT and ALP levels [62]. Additionally, chronic organophosphate exposure in pediatric CKD patients correlated elevated renal injury markers (KIM-1, 8-OHdG) [63], while herbicides like prometryn and atrazine impaired glomerular filtration [64]. Occupational exposure to copper-based fungicides also increased oxidative stress and early renal injury biomarkers (NGAL IGFBP7) in agricultural workers [65]. These findings underscore the diverse mechanisms of pesticide-induced toxicity and the importance of comprehensive biomarker analysis in assessing health risks.

MRP2, an apical membrane-localized transporter in hepatocyte canalicular membranes and renal proximal tubule brush borders, facilitates ATP-dependent efflux transport [66]. It plays a critical role in hepatic function, particularly in bilirubin efflux into bile canaliculi. Drug-induced cytotoxicity can impair MRP2-dependent biliary efflux, contributing to hepatobiliary and renal pathologies [67]. Reduced MRP2 expression is observed in renal insufficiency [68], acute liver failure (ALF) [69], Dubin–Johnson syndrome [70], and cholestasis [71], highlighting its importance in detoxification. Our study evaluated the effects of pesticide exposure on MRP2 expression in 293T and CCC-HEL-1 cells at the half-lethal concentration. Most tested pesticides significantly downregulated MRP2 expression, consistent with its known role in detoxification. Farnesoid X Receptor (FXR), Pregnane X Receptor (PXR), and Constitutive Androstane Receptor (CAR) are known to induce MRP2 expression [72], with PXR particularly crucial in upregulating drug-metabolizing enzymes and transporters like CYP3A4 and MRP2 [73]. This cell-specific response may represent a compensatory adaptation to chemical stress, though the exact molecular mechanisms require further investigation.

## 5. Conclusions

This study delved into the in vitro toxicological mechanisms of eight pesticides by systematically comparing their effects on human and rat hepatorenal cells, identifying chlorothalonil as the most cytotoxic agent, followed by triazole-class pesticides. A critical finding was the consistently greater sensitivity of human cells compared to rat cells. This interspecies divergence serves as key evidence for the distinct xenobiotic metabolism pathways between species, thereby challenging the current reliance on animal data for human toxicity predictions and underscoring the necessity of human-relevant in vitro models in risk assessment. The observed differences in cellular responses point to underlying variations in metabolic enzymes, cellular transport, and stress response pathways. Consequently, our findings highlight the urgent need for multi-omics approaches (transcriptomics, metabolomics) to elucidate these fundamental mechanisms of pesticide-induced toxicity. This research provides a scientific foundation for updating safety standards, developing low-toxicity alternatives, and building more accurate predictive models that account for species-specific biology. Future work should focus on multi-biomarker analyses to decipher pesticide-specific toxicity patterns, ultimately refining risk assessment frameworks and enhancing human health protection strategies.

## Figures and Tables

**Table 1:** IC_50_ values of eight pesticides in four cell lines.

Pesticide	Purity/%	CCC-HEL-1 IC_50_ (mg/L))	IAR IC_50_ (mg/L)	293T IC_50_ (mg/L)	NRK IC_50_ (mg/L)
Myclobutanil	98.0%	455.2	567.0	458.5	588.0
Triadimefon	99.1%	219.2	878.0	106.6	976.8
Propiconazole	98.3%	72.6	188.9	27.2	203.1
Difenoconazole	98.7%	374.0	132.4	208.9	498.0
Tebuconazole	99.7%	134.7	188.1	41.5	171.4
Chlorpyrifos	98.2%	632.7	897.6	444.5	989.5
Chlorothalonil	99.1%	48.1	21.3	32.6	55.6
Pyrimethanil	98.3%	504.8	361.7	111.3	191.6

## Data Availability

The datasets used in this study are available from the corresponding author upon reasonable request. Data will be made available on request.

## Supplementary Materials

The following supporting information can be downloaded at: https://www.mdpi.com/article/10.3390/toxics13110911/s1, Figure S1: IC_50_ values of eight pesticides in rat IAR hepatocytes renal cells; Figure S2: IC_50_ values of eight pesticides in rat NRK renal cells; Figure S3: IC_50_ values of eight pesticides in human 293T cell lines Figure S4: IC_50_ values of eight pesticides in human CCC-HEL-1 cell lines; Figure S5: Cytotoxicity assessment of the DMSO solvent across the concentration range used in pesticide exposures. Table S1: Acute Toxicity and ADI Data for Eight Pesticides. References [74,75,76] are cited in the Supplementary Materials.
