# Supplementary material for: Specific Hepatorenal Toxicity and Cross-Species Susceptibility of Eight Representative Pesticides
Source: Toxics. 2025 Oct 23;13(11):911. doi: 10.3390/toxics13110911 (PMC12656329; doi:10.3390/toxics13110911)
Supplement: Supplementary file 1 [file toxics-13-00911-s001.zip › toxics-3921454-supplementary.pdf]

Supporting Information

Specific Hepatorenal Toxicity and Cross-Species Susceptibility of Eight Representative Pesticides

Yue Liu[a], Ning Xu[b*], Xinyu Song[c], Muchen Deng[c], Ranfeng Sun[a], Peilolong Wang[b], Lidong Cao[a,c*]

1. Pesticide Exposure: After removing the medium, fresh medium containing gradient concentrations (0–2500 mg/L) of each pesticide was added (five replicates per concentration). Following 24 h of exposure, 10 μL of CCK-8 reagent was added to each well.
2. Absorbance Measurement: After 1 h of dark incubation, the OD at 450 nm was measured using a microplate reader.
3. Cell Viability Calculation: Viability was determined by comparing the OD values of treated groups to the blank control.

[a] Yue Liu : Ministry of Education, School of Tropical Agriculture and Forestry, Hainan University, Haikou, Hainan 570228, PR China E-mail address: liuyue2399@163.com

* Ning Xu:Institute of Quality Standard and Testing Technology for Agro-Products, Chinese Academy of Agricultural Sciences, Beijing 100081, China. E-mail address: xuning@caas.cn

* Lidong Cao: Ministry of Education, School of Tropical Agriculture and Forestry, Hainan University, Haikou, Hainan 570228, PR China； Institute of Plant Protection of Chinese Academy of Agricultural Sciences, Haidian District,Beijing 100193,China. caolidong@caas.cn

IAR

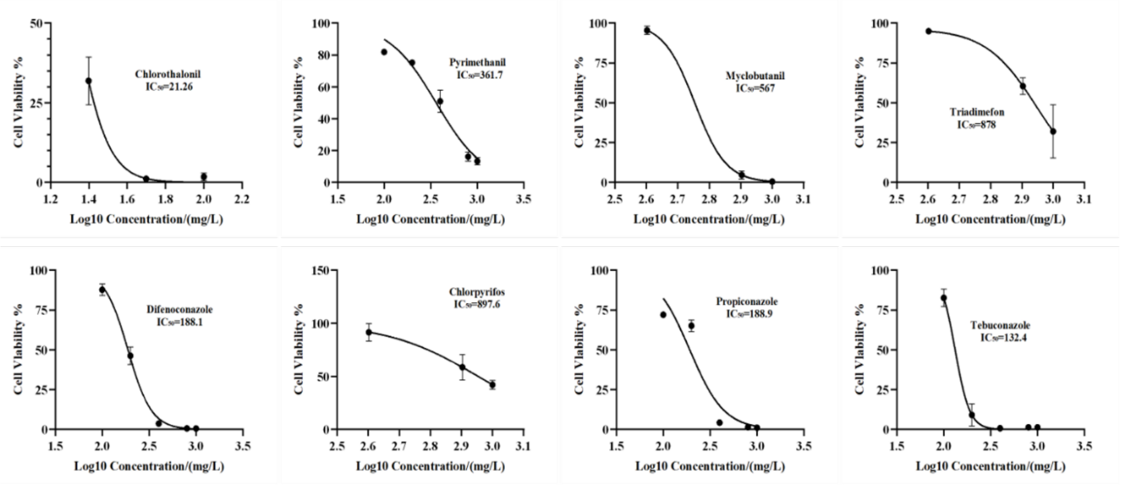

Figure S1. IC_{50} values of eight pesticides in rat IAR hepatocytes renal cells.

NRK

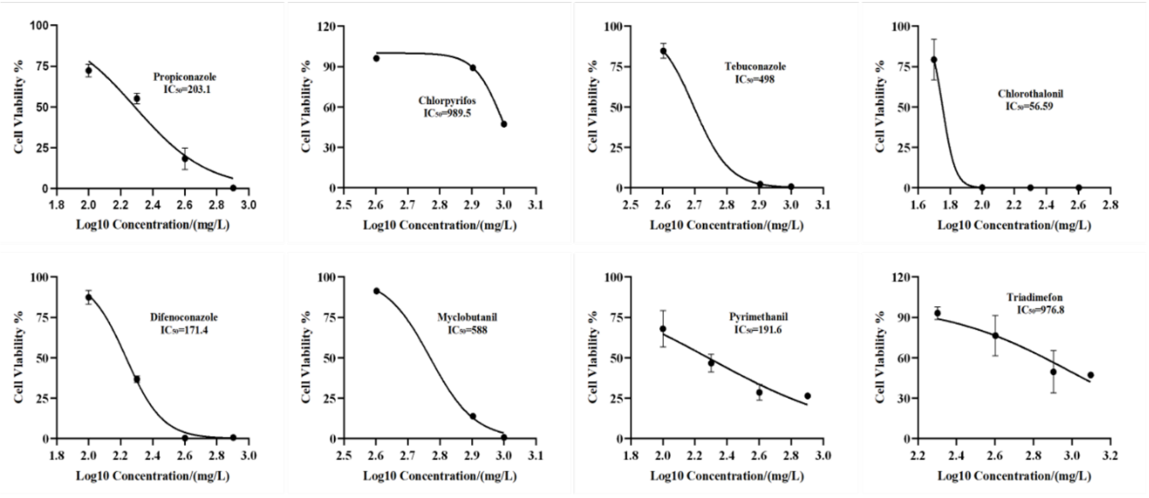

Figure S2. IC_{50} values of eight pesticides in rat NRK renal cells.

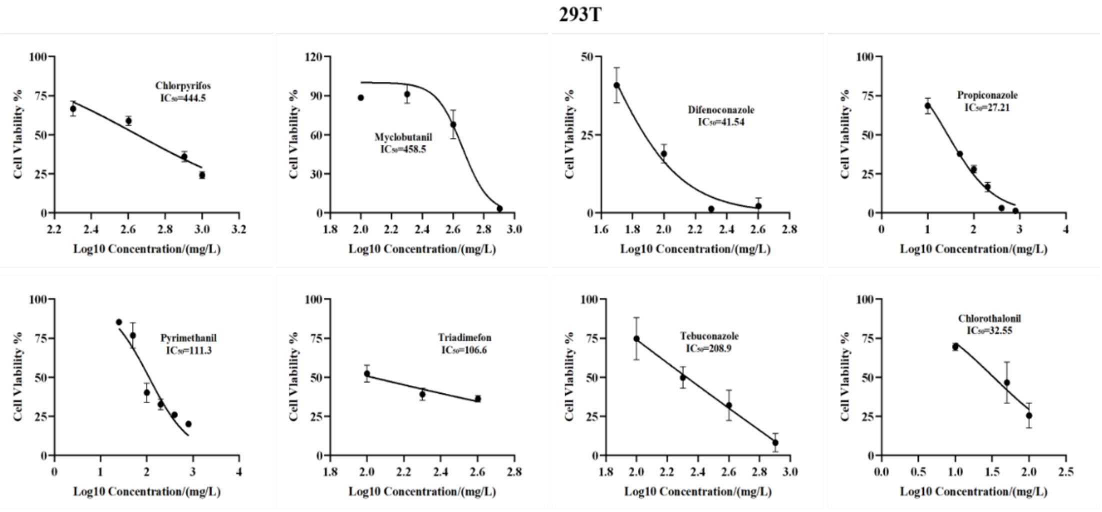

Figure S3. IC_{50} values of eight pesticides in human 293T cell lines.

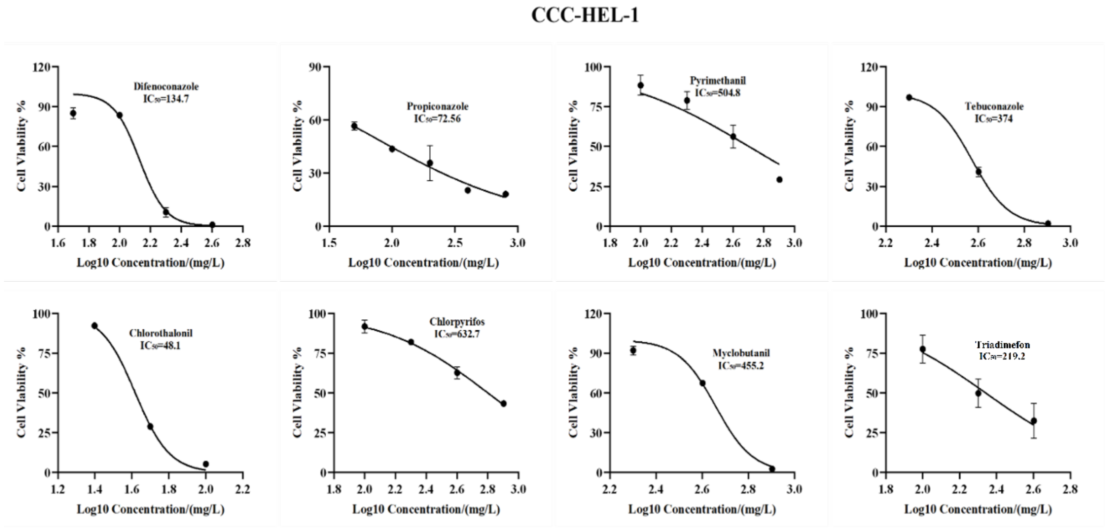

Figure S4. IC_{50} values of eight pesticides in human CCC-HEL-1 cell lines.

The acute toxicity (LD_{50}, LC_{50}) and acceptable daily intake (ADI) values presented in this table were obtained from the PPDB: Pesticide Properties DataBase. The PPDB, developed and maintained by the University of Hertfordshire, UK, is a comprehensive and scientifically validated database that provides detailed information on pesticides. The data can be found at https://sitem.herts.ac.uk/aeru/ppdb/.

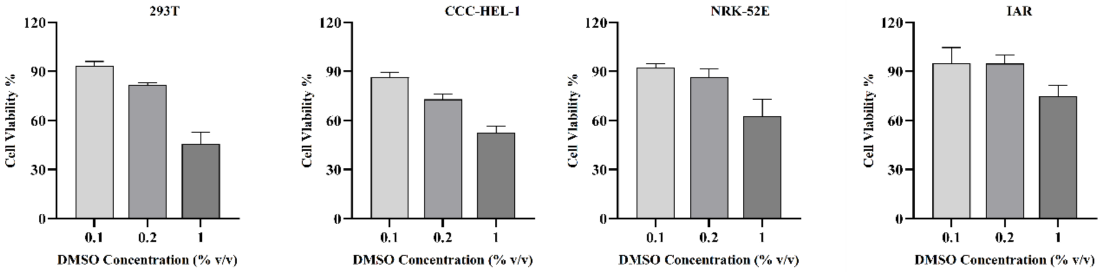

Figure S5. Cytotoxicity assessment of the DMSO solvent across the concentration range used in pesticide exposures.

The figure shows that at DMSO concentrations ranging from 0.1% to 0.2%, the viability of all four cell lines remained stable above 80%.At a 1% DMSO concentration, the viability of all four cell types was around 50% or higher.

Table S1. Acute Toxicity and ADI Data for Eight Pesticides.

	**Mammals - Acute oral LD_{50} (mg kg^{-1})**	**Birds - Acute LD_{50} (mg kg^{-1})**	**Earthworms - Acute 14 day LC_{50} (mg kg^{-1})**	**ADI - Acceptable Daily Intake (mg kg^{-1} bw day^{-1})**	**Toxicokinetic Parameter (Concentration)**
Myclobutanil	1600A5 Rat	510A5 Colinus virginianus	125A5 Lumbricus terrestris corr	0.025 A5 Rat SF=100	Myclobutanil (parent compound): Mean = 38.3 ng/g creatinine [1]
Triadimefon	300G4 Rat	> 2000A5 Colinus virginianus	> 50 P2 Eisenia foetida	0.03 A5A	N/A
Propiconazole	550 A5 Rat	> 2510A5 Anas platyrhynchos	686A5 Eisenia foetida	0.04 A5A	N/A
Difenoconazole	1453A5 Rat	> 2510A5 Anas platyrhynchos	> 610A5 Eisenia foetida	0.01 A5 Rat SF=100	N/A

Tebuconazole	1700A5 Rat	1988A5 Colinus virginianus	1381A5 Eisenia foetida	0.03 A5 Dog SF=100	Tebuconazole-OH (TEB-OH): Concentration range = 0.44 – 0.47 ng/mL[2]
Chlorpyrifos [3]	66 A5 Rat	39.2A5 Colinus virginianus	0.075A5 Eisenia foetida corr	0.001 A5 Rat SF=100	3,5,6-Trichloro-2-pyridinol (TCPy): Highest level = 6.27 ng/mL
Chlorothalonil	> 5000 A5 Rat	> 2000A5 Coturnix japonica	268.5A4 Eisenia foetida	0.015 A5 Rat SF=100	N/A
Pyrimethanil	4150 A5 Rat	> 2000A5 Anas platyrhynchos	313A5 Eisenia foetida corr	0.17 A5 Rat SF=100	N/A

References

74. Ding J, Sun Y, Mortimer M, Guo LH, Yang F. Enantiomer-specific burden of metalaxyl and myclobutanil in non-occupationally exposed population with evidence from dietary intake and urinary excretion. Ecotoxicol Environ Saf. 2023;267:115623.
75. Šulc L, Janoš T, Figueiredo D, Ottenbros I, Šenk P, Mikeš O, et al. Pesticide exposure among Czech adults and children from the CELSPAC-SPECIMEn cohort: Urinary biomarker levels and associated health risks. Environ Res. 2022;214(Pt 3):114002.
76. Huen K, Bradman A, Harley K, Yousefi P, Boyd Barr D, Eskenazi B, et al. Organophosphate pesticide levels in blood and urine of women and newborns living in an agricultural community. Environ Res. 2012;117:8-16.
